# Transition to multiple mini interview (MMI) interviewing for medical school admissions

**DOI:** 10.1007/s40037-020-00605-0

**Published:** 2020-08-24

**Authors:** Tessa Langer, Colby Ruiz, Perry Tsai, Ursula Adams, Camilla Powierza, Ammu Vijay, Paul Alvarez, Gary Beck Dallahan, Lisa Rahangdale

**Affiliations:** 1 University of Illinois at Chicago College of Medicine, Chicago, IL USA; 2 University of North Carolina School of Medicine, Chapel Hill, NC USA; 3 Yale School of Medicine, New Haven, CT USA; 4 The Ohio State University Wexner Medical Center, Columbus, OH USA

**Keywords:** Admissions, MMI, Multiple mini interview, Interview, Medical school

## Abstract

**Introduction:**

The multiple mini interview (MMI) has been incorporated into the holistic review process in the selection of students to US medical schools. The MMI has been used to evaluate interpersonal and intrapersonal attributes which are deemed as necessary for future physicians. We hypothesized that there would be little difference in overall MMI evaluation data compared with traditional interview ratings.

**Methods:**

The University of North Carolina School of Medicine developed an interview process that included a traditional interview and MMI format during the 2019 admissions cycle. Evaluation data along with key demographic variables for 608 MD program applicants were analyzed using descriptive and inferential statistical analyses.

**Results:**

The MMI format slightly favored female over male applicants (*p* = 0.002) but did not select for or against applicants based on age, race/ethnicity, underserved/rural area upbringing, or indicators of disadvantage. Out of 608 applicants, 356 (59%) completed a post-interview survey in which the experience was positively rated.

**Discussion:**

Based on our experience, the use of a hybrid model of traditional interviews complemented with MMI stations provided greater details in the assessment of medical school applicants while obtaining equivalent data and acceptability amongst applicants.

## Introduction

The Association of American Medical Colleges (AAMC) recommends a holistic review process in the selection of students to US medical schools. Holistic review refers to “mission-aligned admissions or selection processes that consider a broad range of factors—experiences, attributes, and academic metrics—when reviewing applications” [1]. The selection process consists of prescreening applications by taking into consideration academic metrics, personal statements, letters of recommendation, and an inventory of extracurricular activities. From this pool, applicants are invited for interviews to assess humanistic characteristics that include interpersonal and leadership skills.

In order to better assess relational skills, many medical schools have shifted from the traditional unstructured interview format towards the multiple mini interview (MMI) [2]. The MMI involves applicants rotating through a series of stations with specific prompts that are designed to assess interpersonal and intrapersonal qualities such as communication, teamwork, and ethical values. MMIs may be a superior method of screening for successful medical students based on interpersonal skills, which have been shown to have a positive association with clinical clerkship grades [3, 4]. Additionally, this method also helps offset the effect of interviewer bias by integrating the assessments of an applicant by multiple interviewers [5] and has been shown to have better reliability compared with traditional interview formats [6].

As medical schools strive to train a diverse physician work force, adopting the MMI format may facilitate this effort. Training a diverse work force is integral to addressing health care disparities [7] and results in more physicians choosing to practice in underserved communities [8]. Because the MMI provides additional metrics about interpersonal and intrapersonal skills, admissions committees can consider other characteristics besides grade point average or standardized test scores. Therefore, applicants who exhibit the desired qualities in a physician but have lower standardized test scores may benefit from this type of interviewing [9]. It should be noted that a small number of studies did report applicants from certain ethnic and social backgrounds did less well [10].

Prior to the fall of 2018, applicants underwent two traditional interviews. Performance on these interviews combined with a review of application materials were used to make decisions for selection by the admissions committee.

In the fall of 2018, our large, public School of Medicine transitioned from traditional interviews to a hybrid system of traditional interview and MMI called ‘Experience UNC’ in order to derive the benefits of both methods [11]. Because this was a new admissions interview process for our institution, our objectives for this study were to ensure interview evaluation metrics were comparable across key demographic variables and to determine if this hybrid admission interview process was well received by applicants.

## Methods

### Study population

This was a cohort study of all interviewed applicants to the University of North Carolina (UNC) School of Medicine during the 2019 admissions cycle. Background information and interview evaluation score averages were collected after the interview season concluded. At the end of the interview day, applicants were emailed a link to a Qualtrics survey about their impressions of the day. The survey was set up to be completely anonymous using Qualtrics settings. This study was approved by the UNC institutional review board (IRB No. 18-3453).

Demographic data were extracted from the American Medical College Application Service (AMCAS) application and included age, gender identification, and race/ethnicity. Applicants were coded as underrepresented in medicine (URM) if they identified as Hispanic (all races), black/African American, American Indian or Alaskan Native, Native Hawaiian or other Pacific Islander.

Rural designation was based on birth county or current county of residence [12]. These counties were classified as Rural and Underserved based on the Area-wide Resource File published by the United States Government, which distinguishes counties by size, degree of urbanization or proximity to metropolitan areas. We identified applicants from underserved areas based on the self-report of their childhood home being from an underserved area, which is defined as ‘inadequate number of available healthcare providers; this may include physicians, nurses, hospitals, clinics, and other healthcare services’’ [12].

We also collected data on receipt of an AMCAS fee waiver, a program based on the applicants’ personal/family/parental income, which must be less than 300% of the 2017 US Department of Health and Human Services’ poverty level guidelines [12].

Information on disadvantaged status was collected, which was based on applicants designating themselves as ‘disadvantaged’ in AMCAS ‘if [they] grew up in an area that was medically underserved or had insufficient access to social, economic, and educational opportunities’ [12]. We also collected data on the AMCAS Socioeconomic Status Disadvantaged Indicator, which designated the applicant as E01 if the parent/guardian had less than a Bachelor’s degree and/or E02 if their occupation was considered service, clerical, skilled, and unskilled labor [12].

### Experience UNC

To prepare for our revised interview days, during the 2017–18 academic year, 15 focus groups were performed with students, educators, administrators, clinicians, and nursing teams at our main School of Medicine campus, regional campuses, outpatient settings and operating rooms. The focus groups were completed during already existing staff or administrative meetings. Each focus group was asked, “What qualities do you think we should be looking for in our medical students?” Themes were reviewed through an iterative process. Seven skills emerged that were consistent with interpersonal, intrapersonal, thinking and reasoning, and science competencies endorsed by the AAMC for entering medical students [13]. Additionally, we consulted with other US schools of medicine and other programs using MMIs. We solicited input from faculty in the Schools of Education and Business on our main campus. After reviewing and synthesizing these findings, six stations were developed using published literature and pilot testing with current medical students. The new interview day, referred to as ‘Experience UNC’, was initiated during the 2018–2019 academic year (2019 admissions cycle).

On the interview day, an overview of the activities was explained to applicants, which consisted of six stations (one traditional interview format and five MMI stations). The traditional interview was performed by a single interviewer, who had access to the applicant’s AMCAS application prior to the interview, and lasted 30 min. A standardized behavioral question based on an interpersonal competency was incorporated into each traditional interview. These are commonly used in the workplace setting by employers to learn how an applicant handled a situation in the past to reveal what skills were used to understand how one may perform in the future.

Experience UNC consisted of two group stations (12–14 min) and two one-on-one discussions (8 min each). A fifth station was designed with the assistance from the UNC simulation center that involved standardized patients. This 8‑minute station allowed applicants to get first-hand experience of our educational curriculum in which students interact with simulated patients in order to develop several of the interpersonal skills required to work with patients in the clinical setting.

### Interviewer selection and training

As noted previously, a variety of individuals participated in the interviews. The Admissions Committee is comprised of faculty who are appointed by the dean as well as voted on by the general faculty. Other interviewers, including medical students, volunteer to serve the medical school.

On the day of the interviews, the admissions dean met with all of the interviewers to explain the station they would be evaluating. At that time, interviewers were provided with a paper copy of the evaluation for the station. The interviewers had time to review and ask clarifying questions about how to complete the evaluation. The MMI stations were evaluated by volunteer physicians, educational staff, and medical students who were blinded to the applicants’ AMCAS application.

### Evaluation of interviews

The six stations evaluated seven competencies. All interviewees were assigned a score of 1–5 for both the traditional interview score and each competency. Traditional interviewers received guides outlining expectations for information gathered during the interview day. Ultimately, they assigned a score based on holistic review of the application and interview. Interviewers underwent training in advance of the MMI station activities and were provided with a standardized station-specific rubric for scoring. Scores were assigned based on performance with a 3 indicating that the applicant was ‘suitable for acceptance’ at our institution. Scores less than 3 indicate that the applicant needed more work and scores above 3 indicated superlative performances.

### Evaluation by applicants

After the interview day, each interviewee was sent a link to an 18-item, anonymous evaluation focused on impressions of the interview day. Overall impressions for each activity throughout the day were evaluated. Applicants indicated their level of agreement using a Likert scale (1 = strongly disagree to 5 = strongly agree). Additional open-ended questions were asked, but for the purposes of this study we are only reporting the Likert scale data. The evaluation was administered using Qualtrics (Provo, UT).

### Analyses

To compare performance of the new MMI stations with the traditional interview, MMI station scores were averaged and correlated with traditional interview scores. An overall MMI average (referred to as MMI average) was calculated that included the new stations and traditional interview evaluations, which was used in our analysis of various demographic characteristics. Descriptive and inferential statistical analyses were used to analyze the overall MMI averages and applicant interview evaluations. We used IBM SPSS version 25 (Chicago, IL) for the analyses.

## Results

For the matriculating class of 2019, the School of Medicine interviewed 608 MD program applicants; 55% of the applicants were female. The average age was 23.5; 49% were aged 22–23 years (Tab. 1). All racial categories were represented, and 20% of the applicants self-identified as one of the racial/ethnic groups we classified as underrepresented in medicine (URM). Eight percent of applicants were born in or currently reside in rural areas, and 27% self-reported that their childhood home was in an underserved area with limited access to healthcare resources. Of those interviewed, 8% applied for and were granted the AMCAS fee waiver based on their family income. Sixteen percent of applicants designated themselves as ‘disadvantaged’ in that they had more limited access to social/economic/educational opportunities. Lastly, 17% of applicants had parents with less than a Bachelor’s degree and/or had an occupation in skilled or unskilled labor.

**Table 1:** Demographics of 2019 applicants

		*N* = 606	% of total
*Gender*			
	Female	333	55
	Male	273	45
*Age*			
	19–21	81	13
	22–23	296	49
	24–29	215	35
	>30	14	2
*Minority status*			
	URM	120	20
	Non-URM	477	79
	No answer	9	1
*Birthplace*			
	US	528	87
	Non-US	78	13
*Birth county*			
	Rural	49	8
	Not rural	557	92
*Legal county*			
	Rural	50	8
	Not rural	556	92
*Underserved*			
	Yes	164	27
	No	413	68
	Did not respond	4	1
	Unknown	25	4
*AMCAS fee waiver*			
	Yes	49	8
	No	557	92
*Disadvantaged status*			
	Yes	94	16
	No	512	84
*SES indicator*			
	Yes	101	17
	No	460	76
	N/A or unknown	45	7

**Fig. 1:**
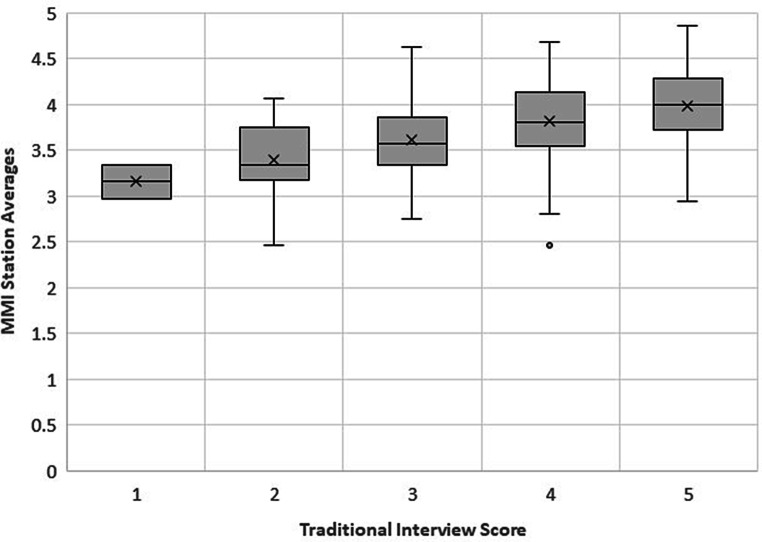
Association between MMI station sverages and traditional interview score

In order to compare the scores for the new MMI stations with the traditional interview format, average ratings from the new MMI stations were correlated with the traditional interviewer score for each applicant. Fig. 1 indicates a positive association between the MMI station average and the traditional interview score. The correlation analysis indicated a fair association [14] between the MMI score and traditional interview (*rho* = 0.357,* p* = 0.001).

MMI average was used to compare applicants based on various demographic variables. The Mann-Whitney U test comparing gender indicated the MMI average score was greater for females (mean = 3.94) than males (mean = 3.84), *U* = 38,905, *p* = 0.002.

Applicant age was not associated with MMI average scores (*rho* = 0.04, *p* = 0.360). In comparing URM designation (mean = 3.89) versus not URM (mean = 3.88), no significant difference was found (*U* = 27,584, *p* = 0.540). An analysis of traditional interview scores by race found no statistically significant differences across race categories. Additionally, applicants from rural counties (*μ* = 3.88) were not different from those from non-rural counties (mean = 3.89; *U* = 13,238, *p* = 0.577). The MMI average score for those from medically underserved areas (*μ* = 3.88) versus those who were not (*μ* = 3.89) was not statistically significant (*t* = −0.27, *p* = 0.785).

The MMI average scores of applicants identified as disadvantaged were not statistically different from those who were not disadvantaged (3.91 vs. 3.89, *U* = 24,659, *p* = 0.703). Candidates with indicators of socioeconomic status disadvantage had comparable MMI average scores (*μ* = 3.87) compared with those without them (*μ* = 3.89; *t* = −0.50, *p* = 0.620).

Tab. 2 provides a summary of applicant evaluations of their interview day. Out of 608 applicants, 356 (59%) completed the survey. Although response rates were strong, we could not compare respondents versus those who did not respond to determine if there was some non-response bias. The applicants rated the experience positively as can be seen by higher scores on positive statements and lower scores on negative statements. To determine if applicants were reading the questions carefully, we looked for inverse relationships for responses. One-tailed Spearman’s correlation was used to compare questions. “*The interview day was well organized*” and “*My time was wasted during the day*” resulted in a *rho* = −0.423, *p* < 0.01. “*The interview process was engaging*” and “*The interview day was too impersonal*” resulted in a *rho* = −0.487, *p* < 0.01. Finally, “*I became well-acquainted with UNC’s curriculum during my interview day*” and “*I left the interview day with unanswered questions about UNC School of Medicine*” resulted in a *rho* = −0.390, *p* < 0.01.

**Table 2:** Applicant evaluation summary

	Mean	SD
The interview day was well-organized	4.07	0.86
The interview process was easy to understand	4.12	0.83
The Office of Admissions staff made me feel welcome at UNC School of Medicine	4.70	0.55
I had enough down time during the interview day	4.48	0.76
My time was wasted during the interview day	1.86	0.88
The lunch session helped me familiarize myself with student life at UNC School of Medicine	3.91	0.90
The Experience UNC session gave me a chance to learn about UNC School of Medicine’s educational environment	3.86	0.96
My one-on-one interviewer gave me (and my application) the attention I deserved	4.53	0.81
I was able to be an active participant in the group sessions	4.38	0.76
The CASPer online assessment added a significant financial burden to the cost of medical school admissions for me	2.20	0.98
The group sessions were uncomfortable	1.94	0.89
The interview day was overwhelming	1.91	0.81
The interview process was unfair	1.70	0.69
The interview process was engaging	4.35	0.61
The interview day was too impersonal	1.99	0.85
I became well-acquainted with UNC’s curriculum during my interview day	3.87	0.92
I left the interview day with unanswered questions about UNC School of Medicine	2.05	0.96
I have a good sense of UNCs educational environment	4.25	0.72

## Discussion

Based on our evaluations of our first year of a new hybrid admissions interview process, traditional interview scores were positively correlated with the MMI station averages, reinforcing evidence supporting MMIs as an interview process. Additionally, finding no statistical difference in the MMI averages across multiple demographic and categorical variables indicates this process did not favor a particular group. The only significant difference for MMI average scores was between genders. Applicants provided overall positive feedback on their interview day experience.

Gender differences on MMI average favored female applicants slightly, which is consistent with prior studies that have evaluated MMI scores within health professional schools. Some have shown females had significantly higher MMI scores compared with males [15–18], while other studies have shown no effect with gender [19]. The variable results in the different studies likely represent inherent differences in the methodology of the MMIs at the individual schools; however, it is an important consideration if these ultimately affect the demographics of a medical school class.

Consistent with our results related to disadvantaged status, a few studies have investigated the effect of MMI scores with the socioeconomic status of the applicant pool and found that there was no significant difference in MMI scores between URM and non-URM applicants. However, indicators of lower socioeconomic status were associated with lower MMI scores [16, 20]. These findings may be in part due to our deliberate efforts to recruit classes of medical students that reflect the population of the state.

The MMI did not select for or against applicants based on age, race/ethnicity, or indicators of disadvantage. We also did not find a difference based on underserved/rural area upbringing. This is contrary to findings from a scoping review of MMI results [10]. This may again be due to the rural nature of the state and the need to train physicians who may go back to those areas of need.

### Limitations

This study involves a single institution and 1 year of data not powered for statistical difference. However, finding little difference in interview evaluations across demographic and categorical variables as well as the positive association with an established form of interviewing can reassure other schools that changing processes is feasible. With the details provided on how we developed this experience, we believe this process could be replicated at other institutions considering implementing an MMI experience for admissions interviews.

As noted previously, due to the evaluation being anonymous we were unable to determine if there was a non-response bias. The link for the evaluation was sent at the end of the interview day, which may have been intimidating for applicants to complete. Additionally, it may have resulted in better evaluations of the experience by those who thought the evaluation was confidential and not anonymous. Future administrations of the evaluation need to carefully consider timing of the evaluation administration with repeated emphasis on its anonymity.

## Conclusions

As the needs of our healthcare workforce change, medical school admissions committees should consider updating how they obtain information for evaluation of applicants. A traditional interview gives applicants an opportunity to highlight unique attributes while the MMI stations allow the admissions committee to get more details about candidates’ skills necessary for future physicians. For our pilot year, we did not analyze MMI station ratings for inter-rater reliability, but will be doing so in the future. Although each medical school must have a process that fits the needs and mission of their institution, use of a hybrid model of traditional interviews complemented with MMI stations may provide greater details in assessment of the evolving pool of medical school applicants.
